# Targeting cancer stem cells expressing an embryonic signature with anti-proteases to decrease their tumor potential

**DOI:** 10.1038/cddis.2013.206

**Published:** 2013-07-04

**Authors:** C Y Darini, P Martin, S Azoulay, M-D Drici, P Hofman, S Obba, C Dani, A Ladoux

**Affiliations:** 1 CNRS, iBV, 28 Avenue de Valombrose, F-06107 Nice, France; 2 University of Nice-SophiaAntipolis, iBV, 28 Avenue de Valombrose, F-06107 Nice, France; 3 Institut de Chimie de Nice UMR 7272, University of Nice-Sophia Antipolis, Parc Valrose, 06108 Nice, France; 4 Pharmacology Department, Nice University Medical Center Hospital, 30 Avenue de la Voie Romaine, 06002 Nice, France; 5 INSERM ERI 21/EA 4319 and Human Biobank, Faculty of Medicine and Pasteur Hospital, CHU of Nice, University of Nice Sophia-Antipolis, 28 Avenue de Valombrose, 06107 Nice, France

**Keywords:** cancer stem cells, HIV-protease inhibitors, stemness signature, apoptosis, Oct-4

## Abstract

Cancer stem cells (CSCs) are a specific subset of cancer cells that sustain tumor growth and dissemination. They might represent a significant treatment target to reduce malignant progression and prevent tumor recurrence. In solid tumors, several hierarchically organized CSC clones coexist, even within a single tumor. Among them, CSCs displaying an embryonic stem cell ‘stemness' signature, based on the expression of Oct-4, Nanog and Sox2, are present in distinct high-grade tumor types associated with poor prognosis. We previously designed a model to isolate pure populations of these CSCs from distinct solid tumors and used it to screen for molecules showing selective toxicity for this type of CSC. Here we show that human immunodeficiency virus (HIV)-protease inhibitors (HIV-PIs) specifically target CSCs expressing an embryonic signature derived from tumors with distinct origins. They reduced proliferation in a dose-dependent manner with a higher specificity as compared with the total population of cancer cells and/or healthy stem cells, and they were efficient in inducing cell death. Lopinavir was the most effective HIV-PI among those tested. It reduced self-renewal and induced apoptosis of CSCs, subsequently impairing *in vivo* CSC-induced allograft formation. Two key pharmacophores in the LPV structure were also identified. They are responsible for the specificity of CSC targeting and also for the overall antitumoral activity. These results contribute to the identification of molecules presenting selective toxicity for CSCs expressing an embryonic stemness signature. This paves the way to promising therapeutic opportunities for patients suffering from solid cancer tumors of poor prognosis.

Cancer stem cells (CSCs) represent a reservoir of self-sustaining cells with a high malignant tumor formation potential. They efficiently form tumors and remain in patients even following conventional therapy.^1^ These cells are not very affected by therapies designed to eliminate rapidly dividing cells, that is, the major component of solid tumors. If the tumor size is reduced by killing the bulk of tumor cells, CSCs can survive and regenerate new tumors.^2^ Any agent able to distinguish between CSCs, the total cancer cell population and, essentially, normal stem cells may constitute the first step toward elaborating more successful cancer treatments. However, identification of such molecules is hindered by the heterogeneity of CSC populations, even within one tumor type^3, 4, 5^ and the availability of pure and well-characterized CSC populations.

Indeed, vast CSC phenotypic diversity has been described in distinct solid tumors based on the expression of cell surface markers such as CD133, CD44,^6, 7, 8, 9^ the activity of enzymes such as ALDH or the ability to exclude cytotoxic molecules.^10, 11^ Co-expression of any of these markers makes it difficult to isolate a sufficient amount of pure CSC populations for drug screening. Furthermore, CSC purification requires a reliable signature, which is somewhat inconsistent with the fickle behavior of these cells.^3, 4^ From a functional standpoint, CSCs are defined by their ability to self-renew, which is essential for their maintenance. This property is also important to indefinitely perpetuate the growth of a malignant cell population^12^ and to recapitulate the hierarchy of the original tumor.^5, 13^ Recently, we used a self-renewal gene tracking strategy to isolate pure populations of CSCs upon *Oct-4* (*pou5f1*) expression. These populations also expressed *Nanog* and *Sox2*, which are all essential genes for the maintenance of ES cells.^14^ Besides their high tumor potential, CSCs expressing an embryonic stemness signature are able to form metastatic tumors mainly in the lungs. This ‘stemness' signature is also found in human tumors associated with tumor dissemination, poor prognosis^15, 16, 17^ and hard to control disease processes.^2^ Like other CSC types, these CSCs are insensitive to most cancer treatments, including chemo- or radiation therapy.^18, 19, 20, 21, 22, 23^ They represent one of several important CSC populations that could be targeted to reduce malignant progression, dissemination and consequently improve the patients' outcome.

Approaches that rely on an alteration of CSC properties are of interest to eliminate this population. As CSCs display an unlimited self-renewal capability governed by proper gene networks, any alteration in their expression may impede tumor development. In our model, disruption of *Oct-4* expression after knockdown using RNA interference impairs self-renewal and is detrimental to both tumor and metastasis developments.^14^ This approach is of great interest but several factors hamper its use *in vivo*. For instance, degradation of small RNAs by enzymes is responsible for poor penetration into tissues.^24^ Targeting CSCs with specific monoclonal antibodies to surface markers such as CD44 was proposed as a powerful approach to treat leukemia^25^ and possibly breast cancer.^26^ Unfortunately, eradication using specific monoclonal antibodies to surface markers was inappropriate as no common cell surface markers have been identified in Oct-4-expressing CSCs.^14^

These limitations prompted us to screen for molecules that could selectively kill CSCs expressing an embryonic signature and enabled us to identify a well-defined class of protease inhibitors (PIs) as candidates.

Human immunodeficiency virus (HIV)-PIs are of paramount importance for highly active anti-retroviral therapy against HIV (highly active anti-retroviral therapy, HAART). HAART was found to clearly improve the quality of life of acquired immune deficiency syndrome (AIDS) patients by lowering the viral charge and increasing the number of CD4-positive T cells, thus contributing to the restoration of the patients' immune system.^27, 28^ In addition to their anti-viral benefits, the efficacy of HIV-PIs to decrease AIDS-associated Kaposi sarcoma^29, 30^ has raised interest in their distinct antitumor properties. They have been shown to efficiently reduce the tumor mass of aggressive neoplasms such as glioblastomas or ovarian cancers,^31, 32^ to reduce xenograft formation from prostatic tumors^33^ as well as the growth of hepatocarcinomas *in vivo*.^34^ However, these studies were performed indiscriminately on total cancer cell populations, thus hindering any identification of PI-specific effects on CSCs, including those expressing an embryonic signature.

In this study, we show that among HIV-PIs, lopinavir (LPV), one of the most widely used HIV-PIs, efficiently distinguishes CSCs among cancer cells and selectively reduces proliferation and self-renewal of Oct-4-expressing CSCs isolated from different types of solid tumor.^14^ Hence, both the total population of cancer cells from the same tumor and healthy stem cells are only affected at higher drug concentrations. Structure–activity relationship (SAR) experiments performed using key intermediates for LPV synthesis led to the identification of essential pharmacophores for LPV-antitumor specificity and activity. LPV-induced death of CSCs was accompanied by activated-caspase 3 (CASP3) expression and cleavage of the DNA repair enzyme poly (ADP-ribose) polymerase, that is, PARP-1, which represents a hallmark of apoptosis.^35^ Finally, *in vivo* treatment of mice with a fixed association of LPV and ritonavir (RTV) resulted in a reduction in allograft formation, indicating a beneficial effect on tumor regression.

Overall, these results indicate that HIV-PIs selectively and potently kill CSCs bearing a high malignant potential and an embryonic stemness signature. This represents a novel and promising approach to directly target this type of cells responsible for tumor growth and cancer relapse.

## Results

### HIV-PIs preferentially decrease CSC proliferation

Proliferation of CSCs and the total tumor cell population was measured in the presence of salinomycin, a potassium ionophore reported to specifically affect breast cancer CSCs,^36^ and of different PIs.

**Figure 1:**
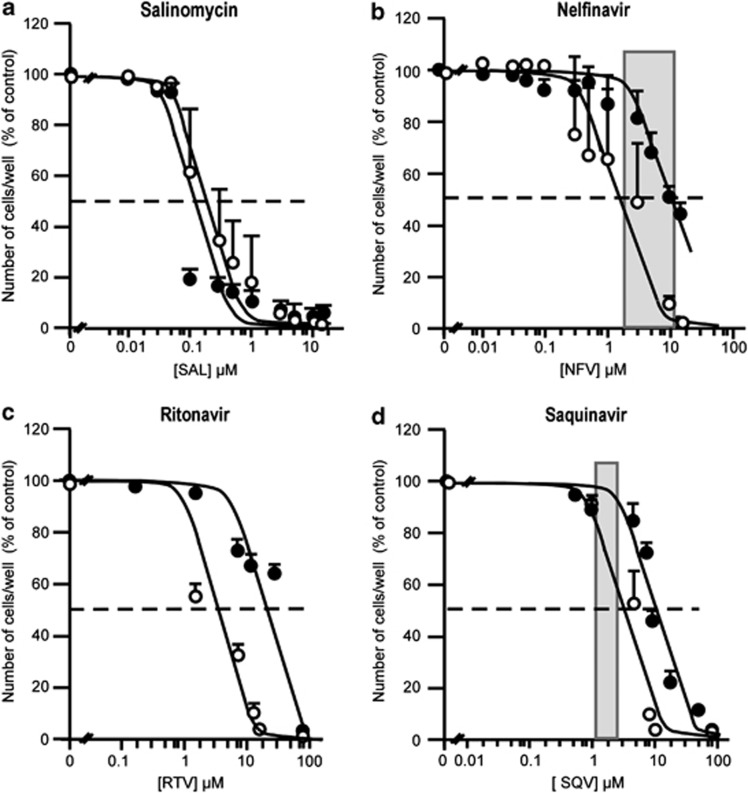
PIs selectively decrease the proliferation of CSCs compared with the total tumor population while salinomycin is efficient on both populations. Dose-response curves for the PI-induced inhibition of cell proliferation for CSCs (open circle) or the total tumor population (closed circles) from an adenocarcinoma in response to the potassium ionophore salinomycin (**a**) and to NFV (**b**), RTV (**c**), SQV (**d**). Grey zones represent the plasma concentrations of the corresponding PI in treated patients, as reported in the literature. The results represent the mean±S.E.M. of three experiments carried out in triplicate. Error bars were omitted when the S.E.M. was smaller than the size of the symbol. IC_50_s were calculated from the curves

Salinomycin reduced proliferation of both CSCs and total population of the same parental tumor with a comparable potency (Figure1a). The range of concentrations corresponds to that reported to efficiently kill breast CSCs. This indicated that salinomycin did not preferentially target CSCs expressing an embryonic signature.

In contrast, among the PIs tested, we found that nelfinavir (NFV), saquinavir (SQV) and RTV were more efficient in reducing CSC growth. The IC_50_s for proliferation inhibition were: 2, 3 and 3.5 μM, respectively, (Figures 1b–d).

Amprenavir (APV) and indinavir (IDV) decreased proliferation of both the total and CSC populations with no selectivity and similar efficacy (IC_50_ in the 10 *μ*M range; data not shown).

**Figure 2:**
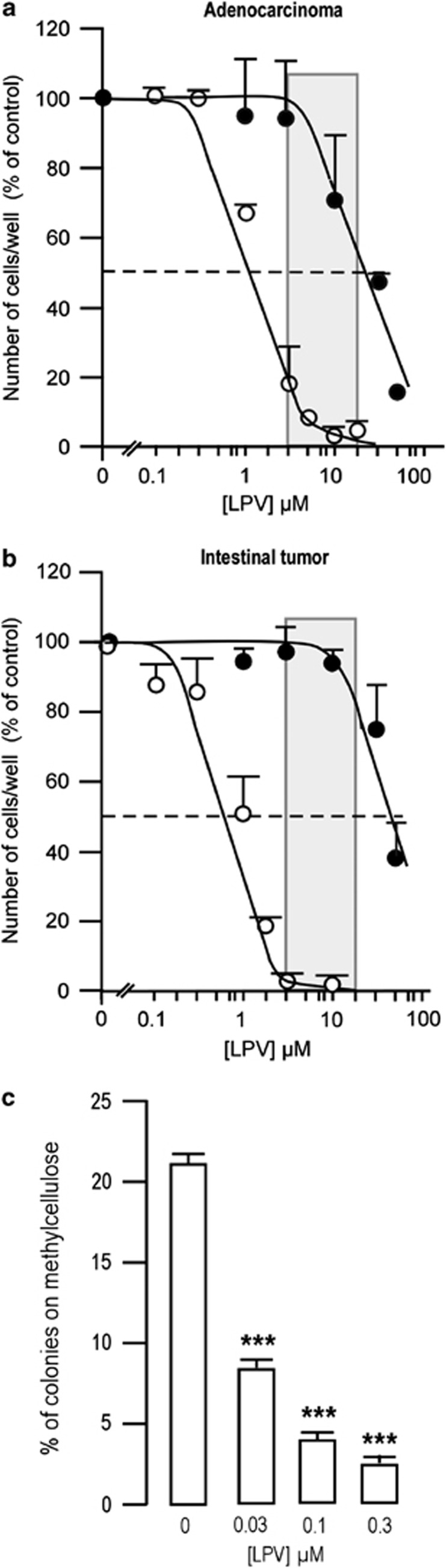
LPV decreases the proliferation and clonogenicity of CSCs. Dose-response curves for LPV-induced inhibition of cell proliferation for CSCs (open circle) or the total tumor population (closed circle) from an adenocarcinoma (**a**), or an intestinal tumor (**b**). Grey zones represent the plasma concentrations of LPV in treated patients, as reported in the literature. The results represent the mean±S.E.M. of three experiments carried out in duplicate. Error bars were omitted when the S.E.M. was smaller than the size of the symbol. IC_50_s were calculated from the curves. (**c**) LPV reduces CSC clonogenicity. After growing 170 cells on methylcellulose for 3 weeks, the percentage of colonies obtained from Oct-4-expressing cells was calculated. Mean±S.E.M. was representative of three independent experiments (***P<0.001)

**Figure 3:**
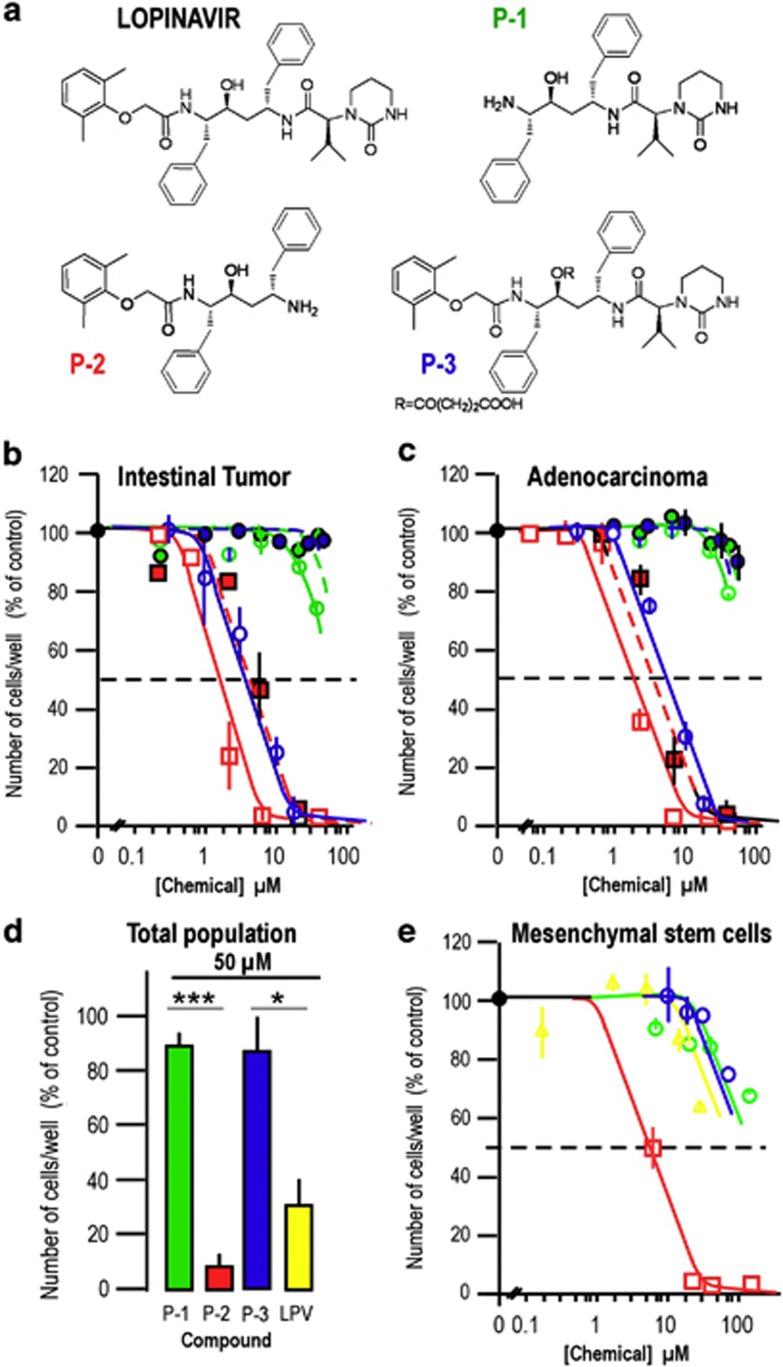
Structure activity of LPV for its antitumoral properties. (**a**) Structures of LPV and pharmacophores. (**b**) Dose-response curves for pharmacophore P-1 (green circles), pharmacophore P-2 (red square) or pharmacophore P-3 (blue circle) -induced inhibition of cell proliferation for CSC (open symbols) or the total population (filled symbols and dashed curves) of an intestinal tumor. The results represent the mean±S.E.M. of three experiments carried out in duplicate. (**c**) Dose-response curves for pharmacophore P-1 (green circles), pharmacophore P-2 (red square) or pharmacophore P-3 (blue circle) -induced inhibition of cell proliferation for CSC (open symbols) or the total population (filled symbols and dashed curves) of an adenocarcinoma. The results represent the mean±S.E.M. of three experiments carried out in duplicate. (**d**) Comparison between the effects of LPV and pharmacophores P-1, P-2, P-3 on cell proliferation of the total population of an intestinal tumor. The results represent the mean±S.E.M. of three experiments carried out in duplicate (*P<0.05; ***P<0.001). (**e**) Dose-response curves for LPV (yellow symbol and curve), pharmacophore P-1 (green circle and curve), or pharmacophore P-2 (red square and curve), or pharmacophore P-3 (blue circle and curve) -induced inhibition of cell proliferation for human mesenchymal stem cells. The results represent the mean±S.E.M. of three experiments carried out in duplicate. For panels (**b**–**e**), error bars were omitted when the S.E.M. was smaller than the size of the symbol

LPV stood out from all PIs and was the most efficient PI affecting the growth of CSCs isolated from two independent tumors (0.6<IC_50_<1.5 *μ*M; Figures 2a and b). The IC_50_s for proliferation inhibition were lower than the therapeutic range of LPV for patients under HAART therapy (2–12 *μ*g/ml, i.e., 3.2–19 *μ*M). For the highest concentrations tested, LPV not only reduced growth but also affected CSC viability as only a few cells remained attached. Depending on the organ from which the tumor was derived, LPV was 25–75 times more efficient in reducing CSC proliferation as compared with the total tumor population (25 *μ*M <IC_50_<50 *μ*M) (Figures 2a and b). Similar results were obtained with healthy mesenchymal stem cells, as LPV impaired their proliferation with IC_50_ >50 *μ*M (Figure 3). In addition, LPV also dose-dependently reduced the clonogenic potential of Oct-4-expressing CSCs, as measured by the percentage of cells able to form colonies on methylcellulose (Figure 2c). This indicated that the decreased proliferation was accompanied by a decreased ability to grow at a single cell level, thus suggesting a loss of self-renewal capacity.

By order of selectivity, PIs can be ranked from the most to the least potent as follows: LPV>NFV>SQV≥RTV>APV=IDV.

### Structure–activity involved in LPV selectivity

As a preliminary SAR study, key intermediates for LPV synthesis (Figure 3a) were prepared and assessed in proliferation assays.

When CSCs were grown in the presence of increasing concentrations of (2S)-*N*-[(2S,4S,5S)-5-amino-4-hydroxy-1,6-diphenylhexan-2-yl]-3-methyl-2-(2-oxo-1,3-diazinan-1-yl) butanamide (or LPV-precursor) **P-1,** no toxic effect was observed on CSCs at LPV concentrations equivalent to or higher than those required to kill the cells (Figures 3b and c). This indicated that the dimethylphenoxyacetic acid moiety was essential for the antitumoral properties of LPV.

In contrast, *N*-[(1S,2S,4S)-4-amino-2-hydroxy-5-phenyl-1-(phenylmethyl)pentyl]-2-(2,6-dimethylphenoxy) acetamide (or LPV-precursor) **P-2**, an intermediate containing the previously identified pharmacophore but without the ℒ-valine carboxylic acid derivative part, displayed efficacy in reducing CSC proliferation in a similar range as compared with LPV. This molecule was also more efficient than LPV in killing the total cancer cell population (Figure 3d), indicating that this chemical group is crucial to distinguish between the two cancer cell populations. Indeed, loss of this pharmacophore induces a high toxicity in human mesenchymal stem cells as compared with LPV, with an IC_50_ of 4–5 *μ*M (Figure 3e).

We then used a modified LPV molecule, referred to as **P-3**, where the central hydroxyl group responsible for anti-protease activity was protected. When CSCs were grown in the presence of increasing P-3 concentrations, a toxic effect was observed at concentrations higher than those required for LPV (IC_50_=6 *μ*M), indicating that the anti-protease activity was essential for the anti-tumor property of LPV (Figures 3b and c). However, P-3 presented a very low (even no) toxicity toward the total cancer cell population (IC_50_ >50 *μ*M, Figures 3 b–d), indicating that the anti-protease activity was greater for toxicity than for selectivity. Moreover, P-3 did not show high toxicity as compared with LPV on mesenchymal stem cells (Figure 3e).

Overall, these results indicate that the anti-proliferative properties of LPV were associated with the presence of the dimethylphenoxyacetic acid moiety and the functional hydroxyl group that is essential for the anti-protease activity, while a distinct part of the molecule was able to selectively target CSCs.

### LPV induces apoptosis in CSCs

**Figure 4:**
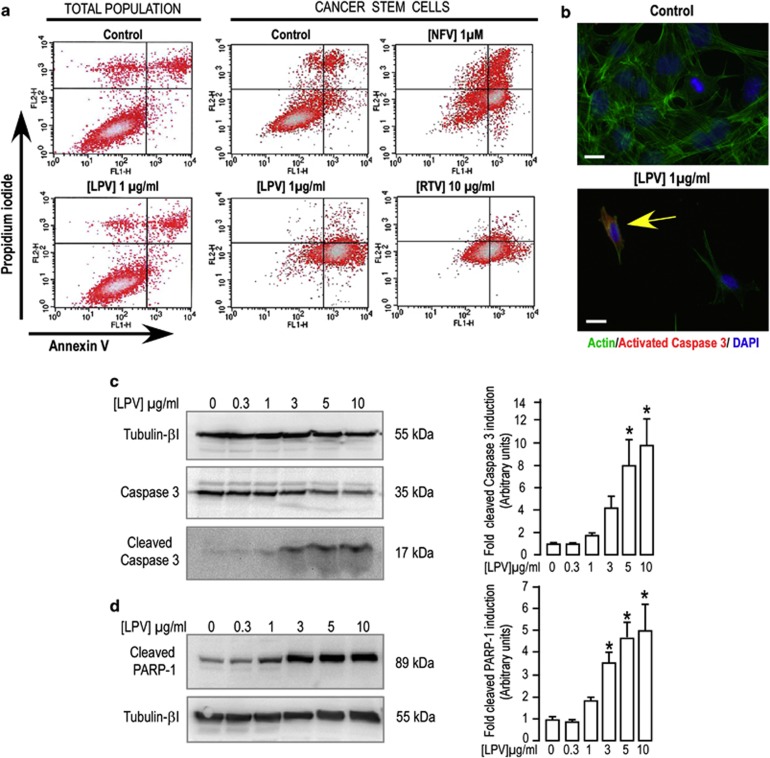
LPV-induced apoptosis in CSCs derived from an adenocarcinoma. (**a**) Total populations of tumor cells or CSCs were incubated with different PIs for 48 h, as indicated and the apoptotic cells were evaluated by flow cytometry with PI/annexin-V double staining. DMSO was used as solvent and as a negative control. The findings of three (total population) or four (CSCs) independent experiments provided confirmations. (**b**) CSCs were exposed to LPV and CASP3 activation was determined 24 h after LPV addition through immunocytochemistry with antibodies against cleaved active CASP3 (17 kDa). Representative images illustrate active CASP3staining (red) in cells following LPV treatment, but cellular red staining is almost absent in cells that did not receive LPV. Phalloidin coupled to Alexa Fluor 488 was used to visualize all cells by means of actin fiber staining (green). Nuclei were labeled blue using DAPI (scale bar, 100 *μ*m). The images are representative of two independent experiments. (**c**) Western blot analysis of CASP3 cleavage in CSCs treated for 24 h with vehicle or increasing LPV concentrations. *β*-Tubulin I is shown as a loading control. These blots are representative of four independent experiments. The histograms represent the expression of cleaved CASP3 normalized to the tubulin signal. Control condition corresponds to cells that did not receive LPV treatment. Mean±S.E.M. obtained from four independent experiments are shown (*P<0.05). (**d**) Western blot analysis of PARP-1 cleavage in CSCs treated for 24 h with vehicle or increasing concentrations of LPV. *β*-Tubulin I is shown as a loading control. These blots are representative of four independent experiments. The histograms represent the expression of the 89 kDa fragment resulting from PARP-1 cleavage normalized to the tubulin signal. The control condition corresponds to cells that did not receive LPV treatment. Mean±S.E.M. obtained from four independent experiments are shown (*P<0.05)

Oct-4-expressing CSCs or cells from the total cancer cell population were treated with individual PIs for 48 h and apoptosis induction was assessed by the loss of plasma membrane asymmetry, as measured by annexin-V-labeling using flow cytometry. A 48-h treatment with LPV (1 *μ*g/ml, i.e., 1.6 *μ*M) induced an increase in annexin-V–fluorescein isothiocyanate (FITC) labeling in 77.5±12.4% (*n*=4) of the cells, while a similar treatment did not induce significant labeling in the total cancer cell population (Figure 4a). In this latter population, 48-h treatment with 20 *μ*g/ml (i.e., 32 *μ*M) LPV was required to significantly increase the percentage of labeled cells to 31±6% (*n*=3) as compared with non-treated cells (data not shown). CSC treatment with NFV (1–3 *μ*M) increased the percentage of annexin-V–FITC-positive cells to 69.2±4.7% and 78.0±5.4%, respectively (*n*=4), whereas higher RTV concentrations (10 *μ*g/ml, i.e., 14 *μ*M) were required to obtain an equivalent result (74.2±10.2% of labeled cells (*n*=6); Figure 4a).

Comparable results were obtained with Oct-4-expressing CSCs derived from another tumor (Supplementary Figure 1A). These results indicate that apoptosis, via annexin-V labeling, could be detected before a decrease in cell proliferation.

Regardless of the CSCs used, this process was associated with CASP3 activation (Figures 4b and c and Supplementary Figures 1B and C) and with PARP-1 cleavage (Figure 4d and Supplementary Figure 1D). Cleaved-CASP3 was detected in cells treated with LPV for 24 h at 1–3 *μ*g/ml concentrations. Its expression increased significantly 10- to 14-fold depending on the cell line, in a dose-dependent manner (Figure 4c and Supplementary Figure 1C). In parallel, LPV treatment induced PARP-1 cleavage in a dose-dependent manner, as measured by the specific accumulation of an 89 kDa fragment in cells (Figure 4d and Supplementary Figure 1D). LPV thus enhanced annexin-V labeling, increased expressions of the activated form of CASP3 and cleaved form of PARP-1 before reducing cell proliferation and altering cell viability.

We next checked if endoplasmic reticulum (ER) stress was involved in this process. We did not note any significantly increased expression of the 78 kDa glucose-regulated protein (also called BiP) after treatment with 5 *μ*g/ml LPV, that is, a concentration able to induce CASP3 cleavage within 24 h (Supplementary Figure 2). Moreover, we did not observe any activation of the pathways downstream of BiP (such as eukaryotic initiation factor 2 phosphorylation or active X-box binding protein 1 synthesis) (data not shown). LPV-induced apoptosis in CSCs thus likely was related to activation of the caspase pathway independently from the activation of ER stress pathways, as reported for other cancer cells.^32^

### LPV reduces CSC-induced tumor progression

We next examined the effects of fixed doses of LPV/RTV (4/1 ratio) administrated in mice, with RTV used to stabilize LPV *in vivo*. This ratio was chosen according to the standard prescription given to treat AIDS patients. We checked that the presence of RTV did not significantly change the IC_50_ for LPV-induced inhibition of proliferation (data not shown). Murine clearance of LPV/RTV was substantial as little if any LPV could be detected in the mouse serum 8-h post-administration.^37^ This prompted us to administer the drugs twice daily, with a minimum interval of 8 h. The LPV/RTV dosage had to be in the range of prescriptions established for humans and appropriate for a long-term treatment of mice. The chosen LPV dose was thus below that recapitulating the drug impregnation similar to the pattern observed in humans^37^ (i.e., causing unwanted secondary effects).

**Figure 5:**
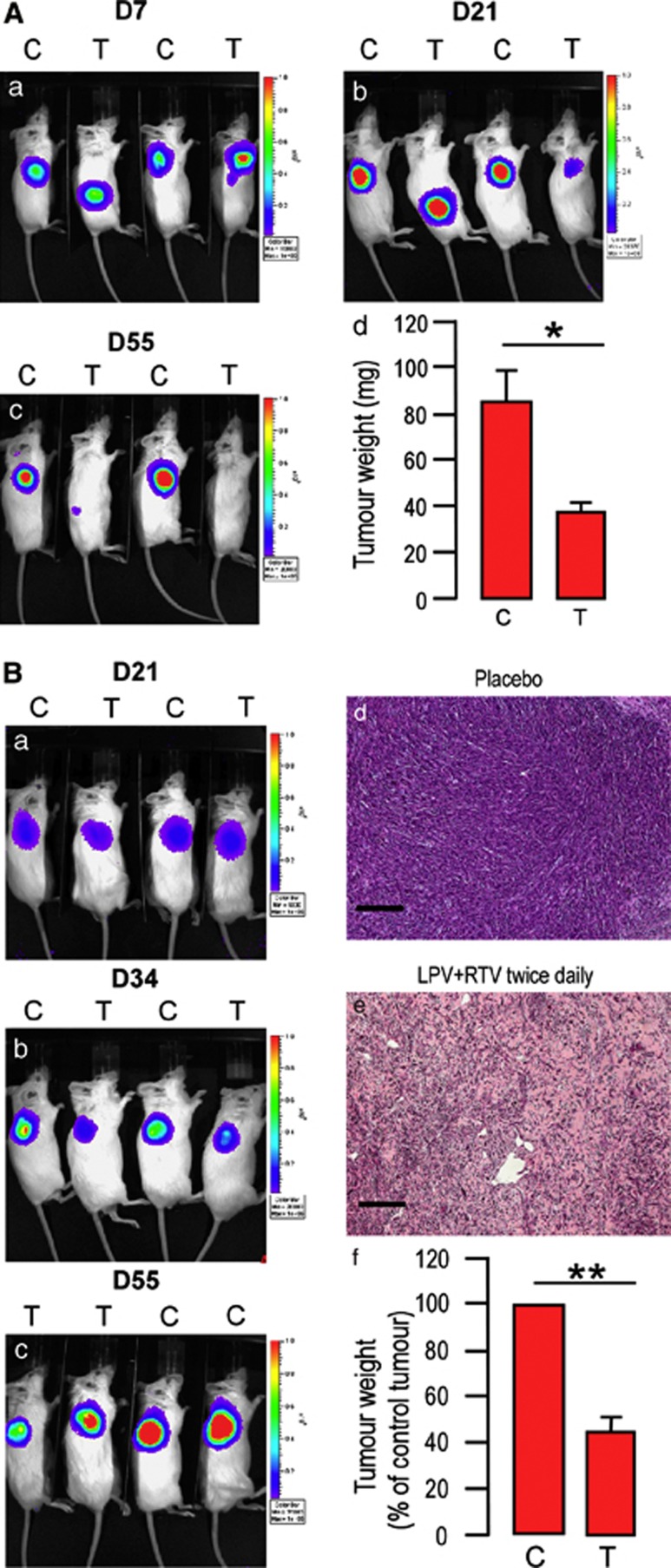
Fixed association of LPV/RTV decreases CSCs-induced allograft formation. CB17/SCID mice transplanted with 250 000 cells from an adenocarcinoma (**A**) or an intestinal tumor (**B**) were treated twice daily with placebo or LPV/RTV. *In vivo* bioluminescent imaging of light emitted by cells reveals a decrease in the size of sites for light emission in mice receiving LPV/RTV after 21 days (Panel A, b) or 34 days (Panel B, b) of treatment, and this was more pronounced after 55 days of treatment. Tumor weight was assessed after 55 days of treatment and was significantly lower in mice receiving LPV/RTV as compared with those receiving placebo (panel **A**, d; panel **B**, f) (*n*=5, *P<0.05; **P<0.01). Histological analysis of the allografts shown in panel **B** (d–e) confirms that LPV/RTV impaired cell proliferation and allograft formation (scale bar, 100 *μ*m)

After 7 days of treatment, no significant difference between mice receiving placebo or active treatment was noted for tumor engraftment (Figure 5Aa). Tumor growth began to decrease slightly after 20–30 days of treatment depending on the parental tumor origin, with CSCs derived from adenocarcinomas being more sensitive to the treatment (Figure 5Ab). After 55 days of treatment, allograft development was significantly reduced as compared with the results in mice receiving placebo, irrespective of the parental tumor the CSCs derived from (Figures 5Ac and Bc).

Histological analysis of the allograft sections confirmed these results. Figure 5B(panels d and e) shows that in mice receiving placebo, allografts developed and presented an undifferentiated phenotype, while the treatment actively restrained the efficacy of CSCs to proliferate and form undifferentiated allografts.

## Discussion

Cancer cell populations are organized in a CSC-oriented hierarchy.^13^ They are of paramount importance for tumor development because they tend to disseminate and form metastases. Conventional therapies are efficient in significantly reducing the tumor burden by eliminating the bulk of cancer cells. Hence, new therapies targeting the CSCs are of interest in that they could purge tumors of the highly malignant CSCs population.

Among the multiple types of CSCs that have been identified in distinct solid tumors,^38^ Oct-4-positive CSCs are associated with high-grade and poor prognosis tumors.^15, 16, 17^ We have developed and previously described^14^ a model to study pure populations of these CSCs from different tumor origins. This model was used to screen for drugs able to specifically kill these cells as compared with the total tumor cell population or to healthy stem cells. HIV-PIs were found to be an efficient antitumor therapeutic class because some of them reduced proliferation, clonogenicity and selectively induced cell death in the CSC population, thus restraining CSC-induced allograft formation.

HIV-PIs are administrated to HIV-positive patients as part of HAART.^28^ They are peptidomimetic drugs designed to mimic the peptide bond targeted by the viral protease but not by any other mammalian endopeptidase,^39^ which means they have a good specificity of action with tolerable adverse effects. HAART has been a major step in the management of HIV infection as it has extended patients' lives by both reducing the viral charge and reconstructing the naive and memory T-cell repertoires, thus delaying or reversing the onset of AIDS.^27^

The benefits of HAART were soon noted in AIDS treatment, but also to reduce HIV-associated cancer risk and tumor burden in HIV-infected persons. AIDS patients are more prone to develop certain types of cancers and the antitumoral effect of PIs was first attributed to their efficacy against viruses involved in AIDS-associated malignancies such as Epstein–Barr or Kaposi sarcoma-associated herpes virus.^39^ However, the antitumoral properties of HAART cannot be entirely explained by these effects or by the recovery of normal immune functions. For instance, complete remission of Kaposi's sarcoma was more prevalent in patients treated with PIs than in those treated with HIV nucleoside reverse transcriptase inhibitors,^29^ which suggests that they have their own specific antitumoral properties. Furthermore, IDV treatment of Kaposi's sarcoma in HIV-negative patients reduced basic fibroblast growth factor production and lowered the number of endothelial cells, hence lowering the tumor progression and improving the clinical course.^30^

The antitumoral effects of HIV-PIs have been explored in various models. They reduce the tumor mass of aggressive neoplasms such as glioblastomas or ovarian cancers.^31, 32^ NFV reduces xenograft formation from myelomas^31, 40^ and prostatic tumors.^33^ IDV reduces hepatocarcinoma growth *in vivo*.^34^ These effects are accompanied by the induction of ER stress, which may in turn trigger cell apoptosis.^32, 41^

However, the antitumoral effects of PIs documented in the literature were obtained in total tumor populations and any extrapolation to pure CSC populations would be risky. In close agreement with the previously reported results, we found that NFV was potent in killing Oct-4-expressing CSCs, but the most potent HIV-PI targeting these CSCs was found to be LPV.

LPV was a weak inducer of cell death in the total cancer cell population at concentrations overlapping those efficient for reducing proliferation. This observation is consistent with the effects of LPVs on meningioma cells as they block proliferation through cell cycle inhibition in an AKT-independent manner without induction of apoptosis.^42^ Conversely, our results showed that LPV induced apoptosis efficiently in Oct-4-positive CSCs, as measured by CASP3 activation and PARP-1 cleavage. We did not notice significant ER stress induction in Oct-4-positive CSCs within the range of concentrations inducing apoptosis, indicating that other mechanisms than ER stress might be involved, as previously reported.^42^

This effect was independent of the parental tumor, but relied much more on the CSC type, as equivalent responses were obtained in Oct-4-expressing CSCs derived from an adenocarcinoma or intestinal tumor. In contrast, salinomycin, which preferentially targets breast cancer CSCs,^36^ reduced the proliferation of both Oct-4-positive CSCs and the total tumor population with comparable potency and efficacy. This indicated that, although active, this molecule did not distinguish between the two cancer cell populations. Our results, along with those reported previously,^43^ confirmed the high potential of this molecule for targeting CSCs independently of an embryonic signature expression. Overall, these observations strongly suggest that efficient targeting of CSCs will require molecules specific to the type of CSCs involved.

However, intracellular pathways leading to the collapse of distinct CSC populations upon drug interaction need to be identified in order to be able to develop appropriate treatments. The mechanism by which salinomycin induces specific breast cancer CSC toxicity^36^ remains unclear. Although being efficient in reducing allografts derived from Oct-4-expressing CSCs, there is no evidence yet that *oct-4* or *nanog* or any other genes contributing to expression of the embryonic signature are potential direct targets for LPV. Other possible targets might be genes whose expressions are regulated by this signature.

The SAR study revealed that the anti-protease activity may be involved in the antitumor activity of LPV. LPV inhibits the HIV protease, that is, a distinct aspartic protease. This enzyme family occurs in higher vertebrates and has been the focus of enormous interest because of the significant roles of these enzymes in human diseases such as hypertension and Alzheimer's disease.^44^ Among them, cathepsin D is highly expressed in cancer cells and associated with metastasis progression.^45^ LPV has been described to exert its antiviral activity with an EC_50_ of 0.1 *μ*M^46^ and to have a higher (>10^5^-fold) specificity for HIV protease as compared with the mammalian aspartic proteinases renin, cathepsin D and cathepsin E.^47^ It is therefore unlikely that the antitumoral activity of LPV results from an interaction with cathepsin D as the IC_50_ measured here was in the 1 *μ*M range (i.e., a lower concentration than that efficient to inhibit mammalian aspartic proteases). Furthermore, we did not note any preferential expression of cathepsin D in CSCs (data not shown). Hence, these types of protease appear to be poor candidates for mediating LPV antitumoral properties, albeit the putative inhibition of a yet to be identified aspartic protease cannot be ruled out.^39^ The decrease in anti-protease activity did not modify the selectivity of LPV toward CSCs. Unfortunately, identification of two pharmacophores of the LPV structure crucial for antitumor potency and selectivity did not allow characterization of an intracellular-specific cascade.

The direct intracellular target(s) of LPV and other HIV-PIs in CSCs, cancer cells and healthy stem cells remain to be determined. Several unwanted side effects have been observed in patients under HAART, including lipodystrophy, insulin resistance and, consequently, diabetes,^48, 49^ yet no single intracellular cascade has been described. Although HIV-PIs inhibit differentiation of normal mesenchymal stem cells,^50^ they also alter the mitochondrial function in different cell types,^51, 52^ indicating that several pathways may be involved, for example, different yet to be identified intracellular pathways involved in LPV antitumor activity. In this regard, the preliminary SAR study generated useful information for designing selective and more potent CSC inhibitors.

Since they are heterogeneous and unstable, new distinct therapies are needed to target the different types of CSC. They would have the advantage of providing personalized cancer treatments that account for both the genetic alterations and CSC status within a tumor, whereas protecting healthy tissues and cells. Overall, our results highlighted that LPV and its derived molecules are promising candidates to selectively reduce the progression of tumors driven by CSCs expressing an embryonic stemness signature. This study represents the first step in the identification of the intrinsic antitumoral properties of HIV-PIs on CSCs. Further studies are needed to decipher the molecular mechanisms underlying HIV-PI antitumoral properties and to design more efficient molecules to wipe out this CSCs population.

## Materials and Methods

### Reagents

Unless specified, all reagents were obtained from Sigma (Saint-Quentin Fallavier, France).

Tissue culture media were obtained from LONZA (Levallois-Perret, France) and fetal calf serum from Dutscher SA (Brumath, France). PIs were obtained by extraction from commercially available tablets and capsules. Their purity was assessed by ^1^H and ^13^C nuclear magnetic resonance and mass spectroscopy. The key intermediates used for SAR studies were synthesized according to a previously reported procedure.^53^

### Mouse model and cell culture

CSCs expressing Oct-4 were isolated from murine tumors that developed in *p53−/−* mice expressing GFP and a puromycin resistance gene under the direction of regulatory sequences of the mouse *Oct-4* gene, as previously reported.^14^ Briefly, CSCs isolated from an adenocarcinoma and an intestinal tumor, were used in this study. Tumor cells were obtained by gentle mechanical dissociation after digestion in the presence of collagenase (0.4 mg/ml Roche Diagnostics, Meylan, France). They were grown under the conditions reported for growing murine ES cells^54^ at 37 °C in a humid atmosphere in the presence of 5% CO_2_. They were isolated after selection with puromycin (1 *μ*g/ml). The cultures were split at the cell growth log phase to prevent overpopulation-induced cell death, and the cells were maintained as previously described.^14^ These CSCs displayed similar properties although they were obtained from two different types of tumor. They expressed other embryonic genes such as *Nanog* and *Sox2*, and their tumor potential was driven by Oct-4 expression.^14^

For proliferation experiments, cells were seeded at 60 000 cells per well in 12-well plates. After 24-h incubation, HIV-PIs were added to the wells and cells were further incubated for 72 h.

Human mesenchymal stem cells were maintained as previously described.^55^ They were seeded at 30 000 cells per well in 12-well plates and treated with LPV or pharmacophores for 72 h.

### Clonogenic assay

CSCs mixed in 5/6 Methocult GF 3434 (STEMCELL Technologies, Grenoble, France) plus 1/6 complete culture medium (v/v) were plated onto petri dishes in the presence or absence of LPV. Colonies (>50 cells) were scored after 30 days of incubation at 37 °C in a humidified atmosphere containing 5% CO_2_, according to the manufacturer's instructions.

### Annexin-V-labeling analysis

Cells were dissociated and allowed to adhere to tissue culture dishes for 24 h. They were then treated with various concentrations of the different PIs for 24 or 48 h and stained with annexin-V coupled to FITC (Life Technologies SAS, Saint-Aubin, France) and propidium iodide (50 *μ*g/ml). Analysis of annexin-V–propidium iodide staining was performed by fluorescence-activated cell sorting (FACS) using a FACS Calibure (BD Biosciences, Le Pont de Claix, France).

### Immunocytochemistry analysis

Cells grown on coverslips were washed with PBS and fixed with Roti-Histofix (Roth, Lauterbourg, France) for 15 min. Fixed cells were incubated in a PBS solution containing Triton X-100: 0.1% (v/v) for 20 min, and treated with PBS containing normal goat serum (5% v/v) for 30 min. Incubation with anti-activated CASP3 antibody (Cell Signaling Technology-Ozyme, Saint-Quentin en Yvelines, France) was carried out at 4 °C overnight followed by incubation with antibody coupled to a red fluorophore, as indicated in the figure legend and phalloidin coupled to Alexa-Fluor 488 (Invitrogen, Cergy-Pontoise, France) to visualize actin fibers. Primary antibodies were omitted for the negative controls. Nuclei were counterstained with Hoechst 33258 (0.5 *μ*g/ml; Invitrogen). Coverslips were mounted using gel mount before visualization using an Axio observer Z1 microscope (Carl Zeiss MicroImaging, Inc., Thornwood, NY, USA).

### Western blot

Cells were rinsed in ice-cold PBS and solubilized in stop buffer^48^ in the presence of Complete PI cocktail (Roche Diagnostics).

Sixty micrograms of proteins were resolved by 8% (PARP-1 or BiP analysis) or 15% (CASP3 analysis) SDS–PAGE under reducing conditions and transferred to immobilon-P membranes (Millipore, Molshiem, France). For immunoblotting assays, the detection antibodies were: rabbit anti-total or anti-activated CASP3, mouse anti-murine cleaved PARP-1 (Asp 214; Cell Signaling Technology-Ozyme), mouse anti-KDEL motif (Stressgen, Tebu-Bio, Le Perray en Yvelines, France) to detect BiP^56^ and mouse anti-β-tubulin I (Sigma). They were diluted in Tris-buffered saline (pH 7.6) containing 0.1% Tween 20 and 5% nonfat dry milk, as indicated in Supplementary Table 1. The membranes were incubated with the diluted antibody overnight at 4 °C.

The bound primary antibody was detected by horseradish peroxidase-conjugated secondary antibody and visualized using an ECL detection kit (Millipore).

Chemiluminescence was observed and quantified using a molecular imager ChemiDoc XRS system (Bio-Rad, Marne la Coquette, France). The band intensity was measured using Bio-Rad Quantity One software.

### Effect of LPV on allograft formation

Allograft induction was performed as previously described.^14^ Briefly, single-cell suspensions were prepared in a PBS-Matrigel (BD Bioscience) mixture (v/v) and injected in a 100 *μ*l volume subcutaneously in the back of CB17/SCID mice (Charles River Laboratories, L'Arbresle, France) anesthetized by i.p. injection of ketamine/xylasine (50 and 10 mg/kg, respectively). Then the mice were divided into two groups: one group received an i.p. injection of a placebo (200 *μ*l of an injectable solution of 5% glucose (p/v)); the other group received a treatment consisting of a twice daily i.p. injection of LPV (0.4 mg per mice per injection) and RTV (0.1 mg per mice per injection) dissolved in 200 *μ*l of an injectable solution of 5% glucose (p/v).

This dosage was chosen to be in the range of dosages administered to humans and to avoid both toxicity and negative side effects that might develop upon a long-term treatment of mice.^37^

The mice were monitored twice daily for 55 days and then killed by CO_2_ asphyxiation and their organs were resected and submitted to histological analysis.

Investigations were conducted according to the French and European rules for care and use of research animals. They were carried out under the supervision of certified researchers in accordance with good animal practice, as defined by the French ‘Direction des Services Vétérinaires'.

### Mouse imaging

Bioluminescent imaging of inoculated cells was performed using a Xenogen IVIS 100 Imaging System (Xenogen Biosciences, Cranbury, NJ, USA). Before imaging, CB17/SCID mice were anesthetized in a chamber with a 1.5% (v/v) isoflurane/air mixture and injected i.p. with luciferin (150 mg/kg body weight).

Validation of the data obtained by bio-imaging to assess the extent of allografts was performed after autopsy of killed mice.

### Histological analysis

Histological analysis was performed on tumors fixed in formalin and embedded in paraffin. Sections were stained with hematoxylin, eosin and safran.

### Statistical analysis

The results are shown as mean±S.E.M. with the number of experiments indicated. Statistical significance was determined by *t-*tests or ANOVA using Micrococal Origin 6.0 (Micrococal Software, Northampton MA, USA). Probability values <0.05 were considered statistically significant and are marked with a single asterisk, <0.01 with double asterisks and <0.001 with triple asterisks.

## Abbreviations

AIDS: acquired immune deficiency syndrome
APV: amprenavir
CASP3: caspase 3
CSC: cancer stem cell
ER stress: endoplasmic reticulum stress
ES: embryonic stem
FITC: fluorescein isothiocyanate
HAART: highly active anti-retroviral therapy
HIV: human immunodeficiency virus
IDV: indinavir
LPV: lopinavir
NFV: nelfinavir
PARP: poly (ADP-ribose) polymerase
PIs: protease inhibitors
RTV: ritonavir
SAR: structure–activity relationship
SQV: saquinavir
