# Supplementary figures and images for: Targeting cancer stem cells expressing an embryonic signature with anti-proteases to decrease their tumor potential
Source: Cell Death Dis. 2013 Jul 4;4(7):e706–. doi: 10.1038/cddis.2013.206 (PMC3730396; doi:10.1038/cddis.2013.206)

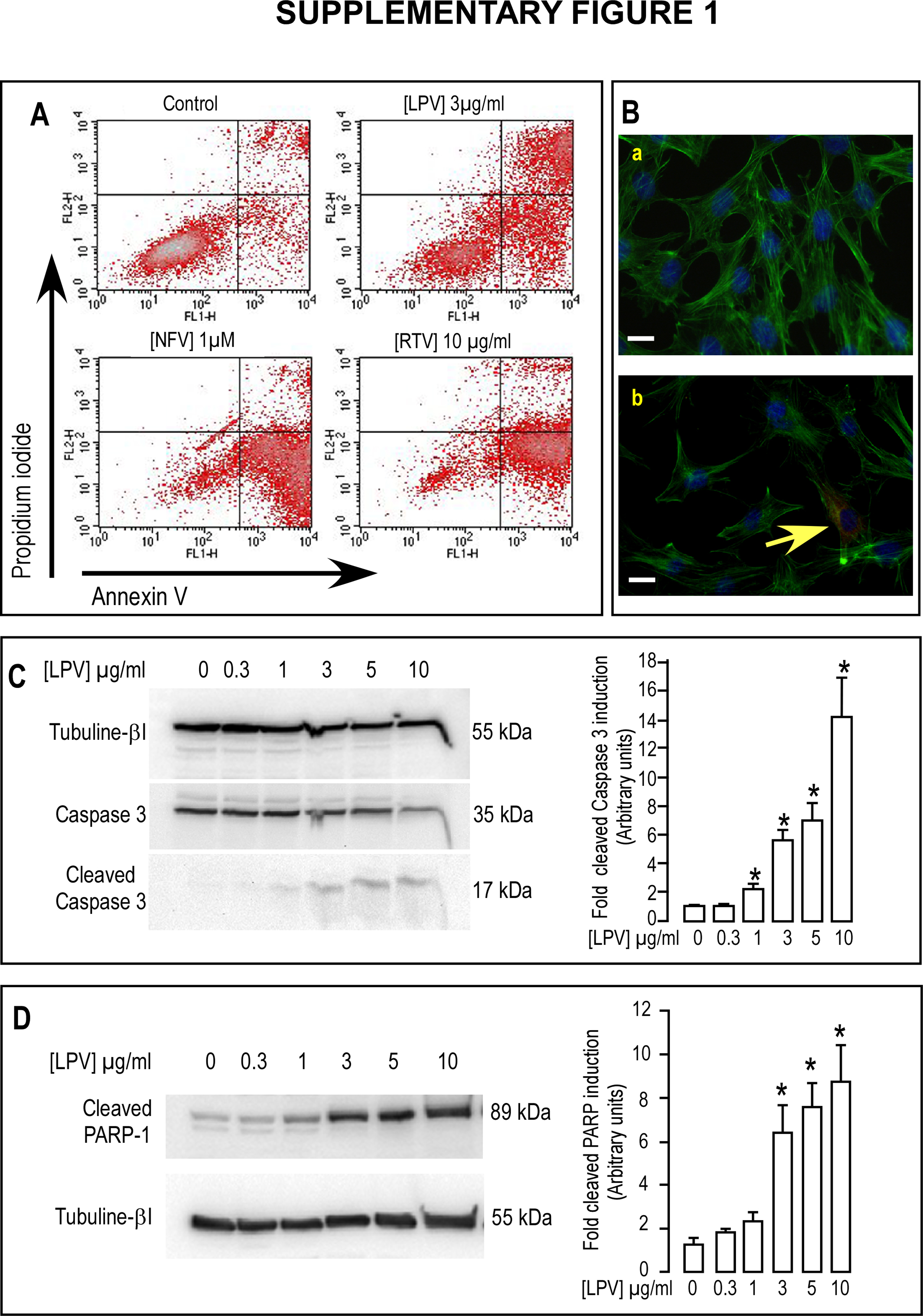

Supplement: Supplementary Figure 1 [file cddis2013206x1.tif]

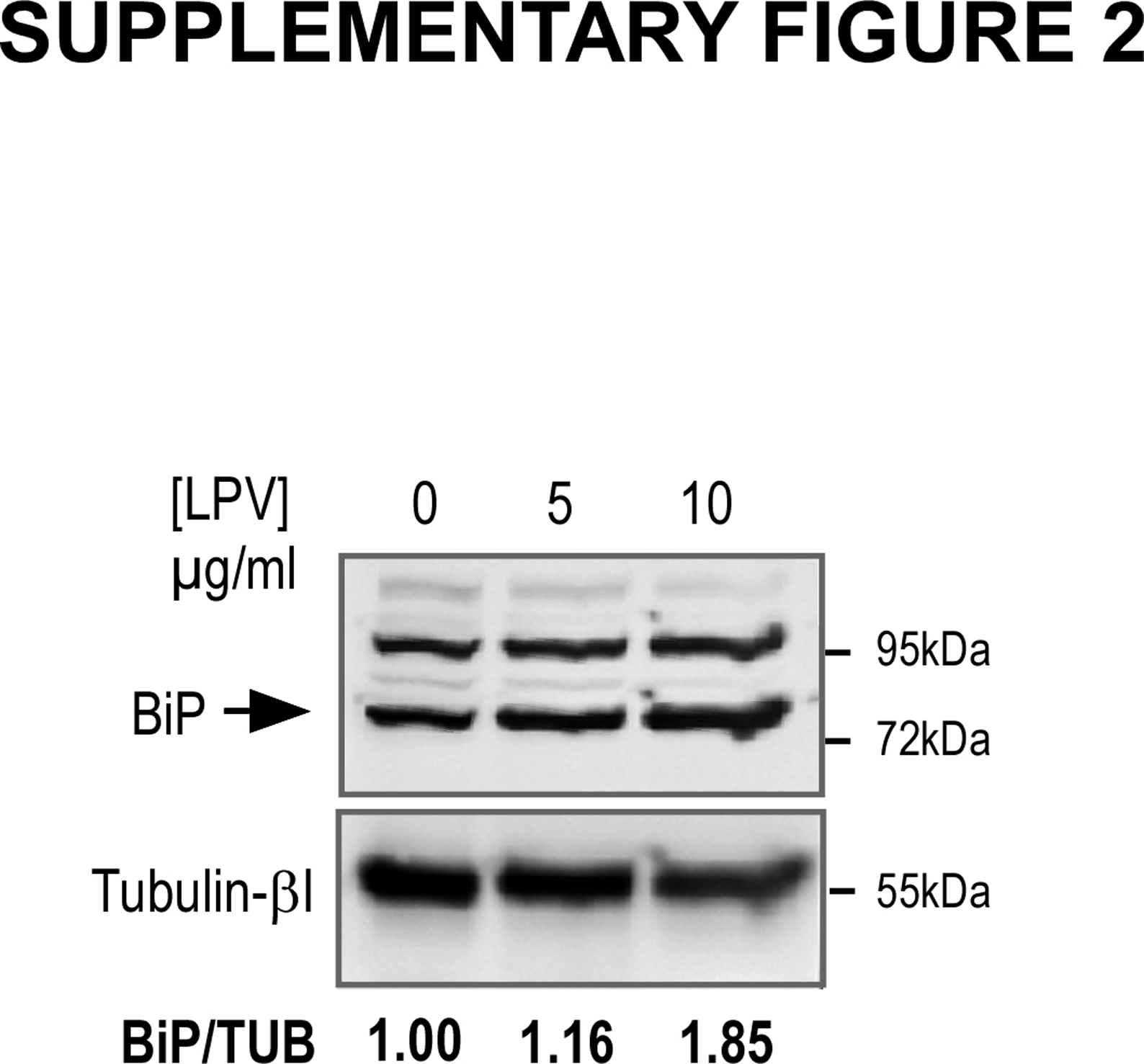

Supplement: Supplementary Figure 2 [file cddis2013206x2.tif]
