# Supplementary material for: Targeting cancer stem cells expressing an embryonic signature with anti-proteases to decrease their tumor potential
Source: Cell Death Dis. 2013 Jul 4;4(7):e706–. doi: 10.1038/cddis.2013.206 (PMC3730396; doi:10.1038/cddis.2013.206)
Supplement: Supplementary Information [file cddis2013206x3.doc]

***Supplementary Material***

**Supplementary Figure 1: LPV induced apoptosis in CSCs derived from an intestinal tumor.**

A/ CSCs were incubated with different IPs, as indicated, and apoptotic cells were evaluated by flow cytometry with PI/annexin-V double staining. DMSO was used as solvent and as negative control.

The findings of four independent experiments provided confirmation.

B/ CSCs were exposed to LPV and caspase 3 activation was determined 24 h after LPV addition through immunocytochemistry with antibodies against cleaved active caspase 3 (17 kDa). Representative images illustrate active caspase 3 staining (red) in cells following LPV treatment (yellow arrow), but cellular red staining is almost absent in cells that did not receive LPV. Phalloidin coupled to Alexa Fluor 488 was used to visualize all cells by means of actin fibre staining (green). Nuclei were labelled blue using DAPI. Images are representative of two independent experiments.

C/ Western blot analysis of caspase-3 cleavage in CSCs treated for 24 h with vehicle or increasing concentrations of LPV. Tubulin is shown as a loading control. These blots are representative of four independent experiments. The histograms represent the expression of cleaved caspase 3 normalized to the tubulin signal. The control condition corresponds to cells that did not receive LPV treatment. Mean + SEM obtained from four independent experiments are shown (* p< 0.05).

D/ Western blot analysis of PARP-1 cleavage in CSCs treated for 24 h with vehicle or increasing concentrations of LPV. Tubulin is shown as a loading control. These blots are representative of four independent experiments. The histograms represent the expression of the 89 kDa fragment resulting from PARP-1 cleavage normalized to the tubulin signal. The control condition corresponds to cells that did not receive LPV treatment. Mean + SEM obtained from four independent experiments are shown (* p< 0.05).

**Supplementary Figure 2: LPV does not induce BiP expression in CSCs derived from an adenocarcinoma.**

Western blot analysis of BiP expression in CSCs treated with increasing doses of LPV for 24 h. Tubulin is shown as a loading control. These blots are representative of two independent experiments and the means calculated are indicated below each condition.

**Supplementary data: Table 1**

| ANTIBODY | MANUFACTURER | CATALOG NUMBER | DILUTION |
| --- | --- | --- | --- |
|  |  |  |  |
| ANTI-CASPASE 3 | Cell Signaling Technology | #9664 | 1000 |
| ANTI-CLEAVED CASPASE 3 | Cell Signaling Technology | #9665 | 1000 |
| ANTI-CLEAVED PARP (Asp 214) | Cell Signaling Technology | #9548 | 1000 |
| ANTI-KDEL motif | STRESSGEN | #SPA-827 | 500 |
| ANTI-β-tubulin I | SIGMA ALDRICH | #T7816 | 5000 |

References for the antibodies used in this study.
